# The MogR Transcriptional Repressor Regulates Nonhierarchal Expression of Flagellar Motility Genes and Virulence in Listeria monocytogenes

**DOI:** 10.1371/journal.ppat.0020030

**Published:** 2006-04-14

**Authors:** Aimee Shen, Darren E Higgins

**Affiliations:** Department of Microbiology and Molecular Genetics, Harvard Medical School, Boston, Massachusetts, United States of America; University of California Berkeley, United States of America

## Abstract

Flagella are surface structures critical for motility and virulence of many bacterial species. In *Listeria monocytogenes,* MogR tightly represses expression of flagellin (FlaA) during extracellular growth at 37 °C and during intracellular infection. MogR is also required for full virulence in a murine model of infection. Using in vitro and in vivo infection models, we determined that the severe virulence defect of MogR-negative bacteria is due to overexpression of FlaA. Specifically, overproduction of FlaA in MogR-negative bacteria caused pleiotropic defects in bacterial division (chaining phenotype), intracellular spread, and virulence in mice. DNA binding and microarray analyses revealed that MogR represses transcription of all known flagellar motility genes by binding directly to a minimum of two TTTT-N_5_-AAAA recognition sites positioned within promoter regions such that RNA polymerase binding is occluded. Analysis of MogR protein levels demonstrated that modulation of MogR repression activity confers the temperature-specificity to flagellar motility gene expression. Epistasis analysis revealed that MogR repression of transcription is antagonized in a temperature-dependent manner by the DegU response regulator and that DegU further regulates FlaA levels through a posttranscriptional mechanism. These studies provide the first known example to our knowledge of a transcriptional repressor functioning as a master regulator controlling nonhierarchal expression of flagellar motility genes.

## Synopsis

Bacteria move in liquid environments using hair-like structures on their surface called flagella. Production of flagella requires more than 40 genes and a significant amount of energy. Indeed, a single flagella filament is composed of approximately 20,000 flagellin subunits. In the environment, bacteria use flagella to swim toward nutrients. Flagella are also important for disease-causing bacteria (pathogens) to initiate infections. However, once inside a person, flagellin is a potent stimulator of the human immune system. Consequently, many bacteria decrease production of flagella shortly after infection. Listeria monocytogenes is a pathogen that grows inside of human cells. In this study, the authors show that L. monocytogenes produce a unique protein (MogR) that downregulates production of all of the genes required to produce flagella. This mode of flagella regulation has not been previously observed in bacteria. The authors demonstrate that in the absence of MogR, L. monocytogenes overproduce flagellin. Misregulation of flagellin production results in bacteria that are no longer able to efficiently enter host cells and are less pathogenic. Thus, the authors demonstrate that MogR-dependent repression of flagellin production is essential for L. monocytogenes to grow inside of host cells.

## Introduction

Flagella are complex surface structures that serve as the primary means of locomotion for many bacterial species and a mechanism by which numerous bacterial pathogens mediate adhesion, invasion, and virulence factor secretion [1]. However, bacterial flagella also activate host immune responses during infection [2]. Thus, flagella production is often repressed following colonization [3,4]. Indeed, flagellar motility gene expression is repressed at physiological temperature in many facultative intracellular pathogens, including Listeria monocytogenes [5–7]. We have recently demonstrated that repression of flagellar motility genes in L. monocytogenes is mediated by a transcriptional repressor, MogR [8]. Although MogR represses transcription of flagellar motility genes at all temperatures, repression by MogR is less stringent at low temperatures to allow for flagella production and motility. Deletion of *mogR* results in elevated transcription of *flaA,* encoding flagellin, relative to wild-type bacteria during broth culture at both room temperature (RT) and 37 °C [8]. Intriguingly, despite producing high levels of *flaA* transcripts during growth at 37 °C, MogR-negative bacteria produce little FlaA protein, indicating that FlaA expression is subject to temperature-dependent posttranscriptional regulation [8]. MogR also represses *flaA* transcription irrespective of temperature during intracellular infection. This environment-specific repression of *flaA* by MogR may be necessary for full virulence, as MogR-negative bacteria exhibit a 250-fold increase in LD_50_ during in vivo infection of mice [8].

In this report, we demonstrate that MogR represses transcription of all known flagellar motility genes in L. monocytogenes and that the severe virulence defect of MogR-negative bacteria is due to overexpression of flagellin during in vivo infection. We also show that MogR directly represses transcription of flagellar motility genes by binding to a minimum of two TTTT-N_5_-AAAA sequences positioned within flagellar motility gene promoter regions such that MogR likely prevents RNA polymerase binding. The ability of MogR to repress gene expression is controlled at the level of MogR activity, as MogR protein levels are temperature independent. Last, we demonstrate that during extracellular growth at low temperatures, MogR repression activity is alleviated by the DegU response regulator, which was previously shown to be necessary for *flaA* transcription [9]. This is the first known example in which modulation of the activity of a master repressor protein controls transcription of all known flagellar motility genes in a bacterial species.

## Results

### Overexpression of Flagellin by MogR-Negative Bacteria Is Deleterious to Infection

We previously showed that a MogR-negative L. monocytogenes strain (Δ*mogR,* DH-L1156) overexpresses *flaA* and exhibits a 250-fold increase in LD_50_ during infection [8]. Since expression of flagellin during infection can compromise the virulence of other bacterial pathogens [3,10], we examined whether overexpression of FlaA specifically attenuates the virulence of MogR-negative L. monocytogenes. To this end, we determined the virulence phenotype of MogR-, FlaA-negative L. monocytogenes (Δ*mogR ΔflaA,* DH-L1248). Loss of FlaA expression in the Δ*mogR* background essentially restored virulence (Table 1, RT), as the LD_50_ for Δ*mogR* Δ*flaA* (1 to 2 × 10^4^) was comparable to that of wild-type (3 to 5 × 10^3^) and approximately 100-fold lower than the LD_50_ observed for Δ*mogR* (approximately 1 × 10^6^). These results indicate that overproduction of FlaA by Δ*mogR* was primarily responsible for the observed 250-fold virulence defect.

**Table 1:**
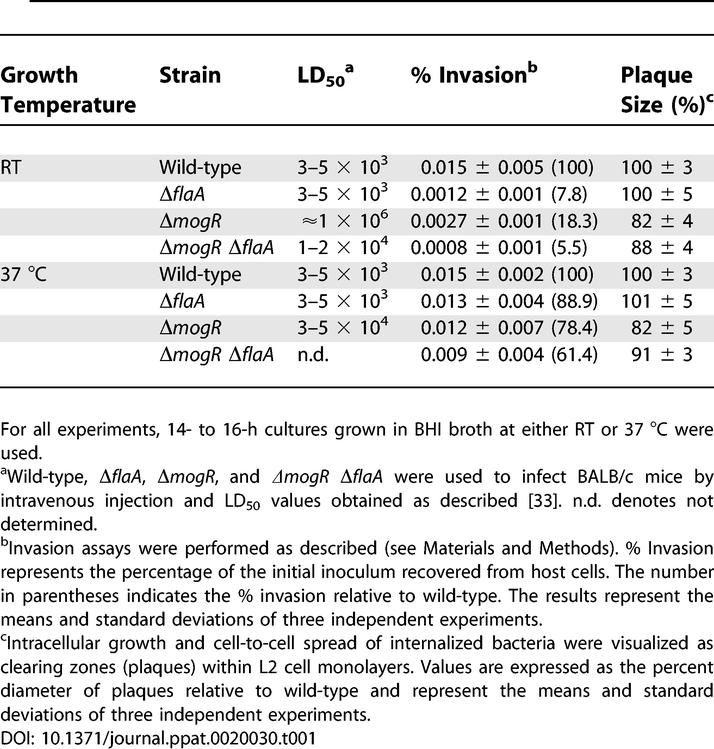
Virulence, Invasion, and Intracellular Spread of L. monocytogenes Strains

**Figure 1:**
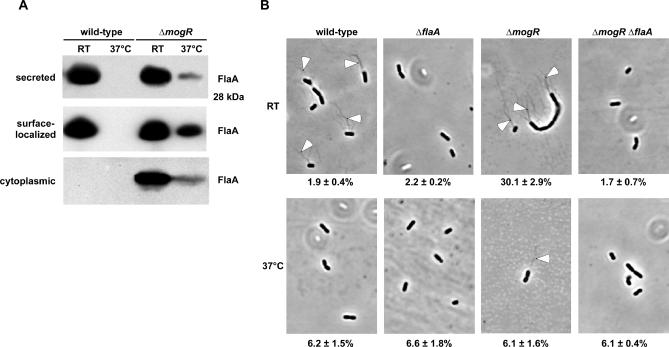
Overexpression of Flagellin by MogR-Negative L. monocytogenes at RT Results in a Chaining Phenotype (A) Western blot analysis of FlaA levels in cellular fractions of wild-type and Δ*mogR* strains. L. monocytogenes cultures were grown at RT or 37 °C for approximately 24 h. Secreted, surface-localized, and cytoplasmic FlaA protein was detected by Western blot using a FlaA-specific antibody. (B) L. monocytogenes strains wild-type, Δ*flaA,* Δ*mogR,* and *ΔmogR* Δ*flaA* were grown at RT or 37 °C for approximately 24 h, stained for flagella, and analyzed by phase-contrast microscopy. Open arrowheads indicate flagella. The percentage of chaining events within a given population is shown below each panel. A chaining event was defined as a chain of three or more bacteria. A total of 300 bacterial events were counted for each population. The mean and standard deviation of three independent experiments are given.

Virulence of MogR-negative bacteria was also found to be dependent on the temperature at which bacteria were cultured prior to in vivo infection, as the LD_50_ value of Δ*mogR* grown at RT prior to infection of mice was 25-fold higher than when Δ*mogR* was grown at 37 °C prior to infection (Table 1). Interestingly, our previous studies indicated that FlaA production in Δ*mogR* was also regulated by temperature [8]. At 37 °C, MogR-negative bacteria produced significantly lower levels of FlaA than when grown at RT (Figure 1A). Given that virulence and FlaA production were both modulated by temperature in MogR-negative bacteria, we hypothesized that overproduction of FlaA by Δ*mogR* during growth at RT might compromise early stages of infection. Microscopic analysis of bacterial cultures revealed that MogR-negative bacteria grown at RT were present in chains at 15 times the frequency observed for wild-type *L. monocytogenes,* whereas no chaining phenotype was observed in Δ*mogR* relative to wild-type when grown at 37 °C (Figure 1B). Deletion of *flaA* in the Δ*mogR* background restored wild-type cell morphology to Δ*mogR ΔflaA* (Figure 1B, RT), suggesting that elevated levels of FlaA, particularly in the cytoplasm of Δ*mogR,* induced the chaining phenotype (Figure 1A and 1B).

Previous studies have shown that L. monocytogenes mutants exhibiting a chaining phenotype are attenuated in virulence due to a defect in host cell invasion and intracellular motility [11,12]. Thus, we examined whether MogR-negative bacteria present in chains were defective in invasion of host cells or cell-to-cell spread. When MogR-negative bacteria were cultured at RT to induce the chaining phenotype prior to invasion of host cells, an 82% decrease in host cell invasion was observed relative to wild-type (Table 1, RT). In contrast, when grown at 37 °C prior to infection, MogR-negative bacteria exhibited only a slight defect (22%) in invasion relative to wild-type (Table 1, 37 °C). As expected, bacteria lacking flagellin expression (Δ*flaA* and Δ*mogR ΔflaA*) were severely impaired in host cell invasion (92% and 94% defect, respectively) compared to wild-type grown at RT prior to infection. This phenotype likely results from reduced host cell association due to a lack of flagellar-based motility [13]. These results associate the chaining phenotype of MogR-negative bacteria grown at RT with a defect in host cell invasion.

MogR-negative bacteria grown at RT prior to infection exhibited an approximately 20% defect in intracellular spread as measured by in vitro plaque formation (Table 1, RT). However, unlike the invasion defect, the plaquing defect was independent of the temperature at which bacteria were cultured prior to infection (Table 1), perhaps because the chaining phenotype of MogR-negative bacteria was not maintained upon intracellular replication (unpublished data). Furthermore, deletion of *flaA* in the Δ*mogR* background only partially alleviated the cell-to-cell spreading defect (Table 1), indicating that overexpression of FlaA alone cannot account for the intracellular spreading defect of Δ*mogR*. Notably, the 10% plaquing defect of Δ*mogR ΔflaA* correlated with the observed approximately 2- to 6-fold virulence defect of these bacteria (Table 1, RT). Thus, these results suggest that overproduction of FlaA by MogR-negative bacteria cultured at RT prior to infection induces a chaining phenotype that primarily impairs host cell invasion and significantly reduces virulence in the mouse model of infection.

### MogR Primarily Functions to Repress Flagellar Motility Gene Expression at 37 °C

**Figure 2:**
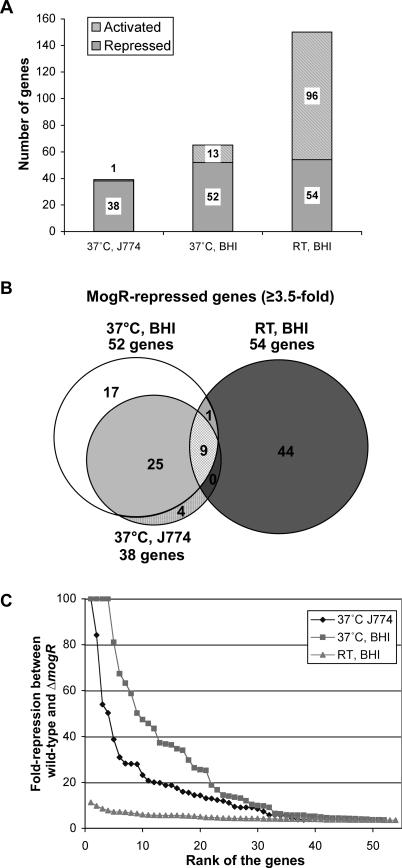
MogR Primarily Functions as a Transcriptional Repressor at 37 °C (A) Graphical representation of genes differentially expressed between Δ*mogR* and wild-type during growth in different conditions as determined by microarray analysis. Only genes whose absolute expression was altered by a minimum of 3.5-fold (*p* < 0.01) are represented. Shaded bars indicate genes repressed by MogR, and hatched bars represent genes activated by MogR. (B) Venn diagram representation of MogR-repressed genes. Only genes that were downregulated in wild-type relative to Δ*mogR* by at least 3.5-fold (*p* < 0.01) are presented. (C) Graphical analysis of the fold-repression of genes regulated by MogR during growth in different conditions. Genes were defined as MogR repressed if they exhibited at least a 3.5 fold-change (*p* < 0.01) between wild-type and *ΔmogR.* The most highly repressed gene in a given condition was assigned a rank of 1. Gene rankings were plotted against fold-repression. The average fold-repression was 5-, 27-, and 21-fold for growth in BHI at RT, BHI at 37 °C, and J774 host cells at 37 °C, respectively.

To potentially identity factors contributing to the intracellular spreading defect of MogR-, FlaA-negative bacteria, we determined the MogR regulon during infection of J774 macrophages at 37 °C. Using microarray analysis, 39 genes were identified as being differentially regulated by a factor of 3.5-fold or greater (*p* < 0.01) between wild-type and MogR-negative bacteria (Table S1). Of these 39 genes, 38 were repressed by MogR (Figure 2A) and located either within the major flagellar motility gene cluster or in an operon with additional flagellar motility genes (Table 2). The single MogR-activated gene was *comC,* a homolog of the *Bacillus subtilis comC* gene that encodes a type IV prepilin peptidase required for competence in B. subtilis. Since L. monocytogenes is not naturally competent, expression of *comC* likely has minimal effect on the virulence of Δ*mogR*. Thus, these results implied that the primary function of MogR during intracellular infection is to repress flagellar motility gene expression.

**Table 2:**
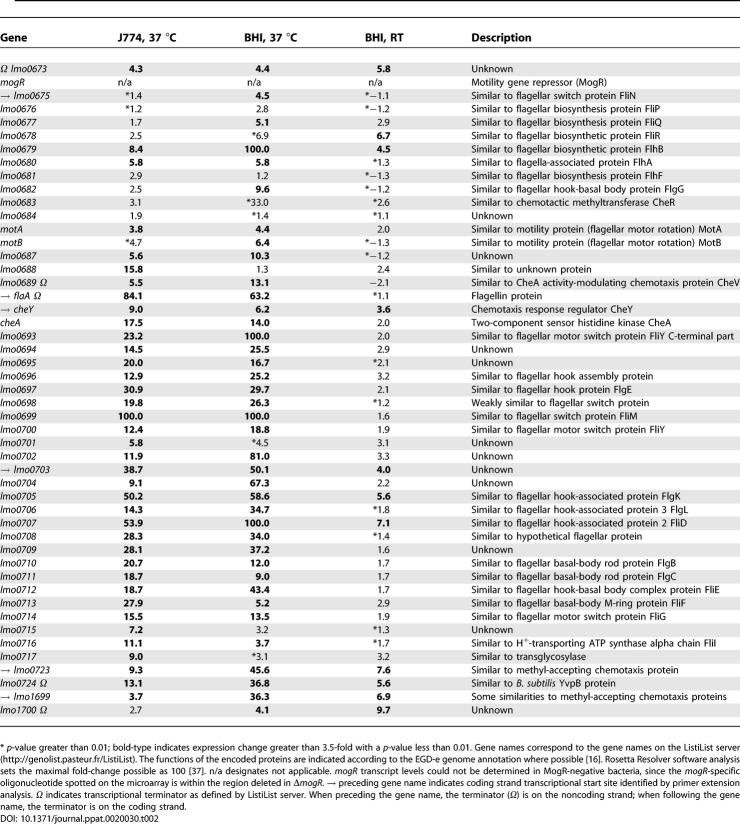
Microarray Analysis of Flagellar Motility Gene Expression in MogR-Negative L. monocytogenes Relative to Wild-Type during Growth in Different Conditions

The microarray results were consistent with prior studies indicating that MogR represses flagellar motility gene expression during extracellular growth at 37 °C. Previously, three flagellar motility genes, *cheY* (encoding the chemotaxis regulator CheY)*, lmo0675* (encoding a flagellar switch protein), and *flaA,* were found to be MogR repressed [8]. To determine whether MogR globally represses flagellar motility gene expression in a temperature-dependent manner and to potentially identify additional MogR-regulated genes, we compared the transcriptional profile of Δ*mogR* to wild-type during growth in brain heart infusion (BHI) broth at both RT and 37 °C. A total of 65 L. monocytogenes genes were identified as being MogR regulated by a minimum of 3.5-fold (*p* < 0.01) during growth in BHI at 37 °C (Table S1), of which 52 genes were expressed at least 3.5-fold higher in MogR-negative bacteria relative to wild-type (Figure 2A). Of these 52 MogR-repressed genes, 39 were flagellar motility genes or located in operons encoding flagellar motility genes (Table 2). Thus, the MogR regulons defined during growth in BHI broth and infection of J774 macrophages at 37 °C were largely identical (Figure 2B), implying that the main function of MogR is to repress flagellar motility genes at 37 °C regardless of whether bacteria are growing extracellularly or within host cells.

In contrast, the transcriptional profile obtained during growth at RT differed substantially from the profile defined during growth at 37 °C (Figure 2B). More genes were identified as MogR activated than MogR repressed when L. monocytogenes was cultured at RT (Table S1). Of the 150 genes that were differentially expressed at least 3.5-fold (*p* < 0.01) in MogR-negative bacteria relative to wild-type at RT in BHI broth (Figure 2A), 96 genes were activated by MogR with an average fold-change of 8.3-fold, whereas 54 genes were MogR repressed by an average of 4.9-fold. This latter value is marginally greater than the minimum 3.5-fold change we used to define a gene as MogR regulated. In contrast, the average fold-change for genes identified as being MogR repressed during growth at 37 °C in J774 cells and in BHI broth was 21-fold and 27-fold, respectively (Figure 2C). Thus, MogR repression activity was reduced during growth in BHI broth at RT relative to 37 °C. Indeed, only nine genes were MogR repressed in all three conditions examined (Figure 2B). Although all nine genes were either related to flagellar motility or located within an operon with additional flagellar motility genes, they were repressed to a much lesser extent during growth at RT in BHI broth (Table 2).

### MogR Binds Directly to the Promoter Regions of Multiple Flagellar Motility Genes

**Figure 3:**
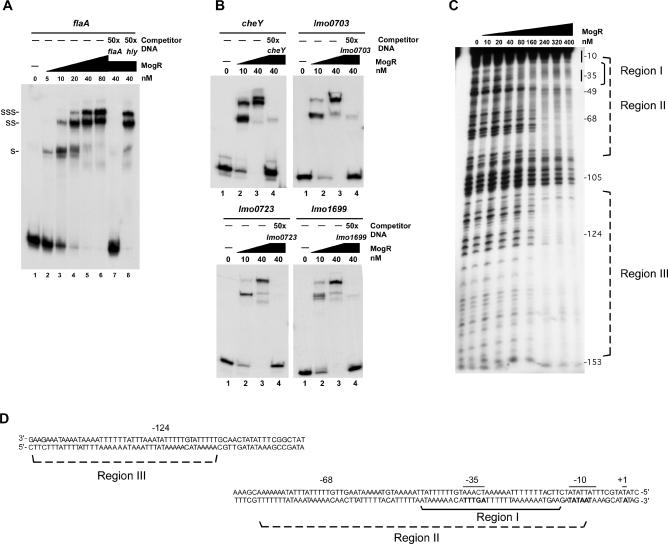
Purified MogR Is Sufficient to Bind the Promoter Regions of Multiple Flagellar Motility Genes (A) Gel shift analysis of MogR binding to the flaA promoter region. Radiolabeled flaA promoter DNA spanning region −162 to +8 relative to the transcription start site was incubated with increasing concentrations of purified His_6_-tagged MogR (lanes 1 to 8) and in the presence of unlabeled competitor DNA (lanes 7 and 8). The binding reactions were analyzed by nondenaturing PAGE. The identity of unlabeled competitor DNA added in 50-fold excess to binding reactions is indicated. Shifted (S), supershifted (SS), and super-supershifted (SSS) DNA complexes are indicated. (B) Gel shift analysis of MogR binding to flagellar motility gene promoter regions. Increasing amounts of purified His_6_-tagged MogR were incubated with various radiolabeled DNA probes (lanes 1 to 4) and in the presence of unlabeled competitor probe DNA of the same identity (lane 4). The identity of each probe is indicated. The probe for *cheY* spans the region from −108 to +74 relative to the transcription start site. The probe for *lmo0703* spans the region from −164 to +21 relative to the translation start site. The probes for *lmo0723* and *lmo1699* span the regions from −123 to +18 relative to the translation start site. (C) DNase I footprinting analysis of MogR binding to the *flaA* promoter region. The noncoding strand probe spans the region from −162 to +8 relative to the transcription start site. Negative numbers indicate the approximate distance from the transcriptional start site. The high affinity MogR binding site is indicated by a solid bracket and is denoted as region I. The lower affinity binding sites are marked with dashed brackets and identified as regions II and III. (D) DNA sequence of the *flaA* promoter region. The bottom strand is the coding strand and reads 5′ to 3′. The −35 and −10 elements and transcription start site (+1) are indicated in bold and by a solid line above the sequence. Solid bracket indicates the region protected at low MogR concentrations (region I), while the dashed brackets mark the regions protected with higher concentrations of MogR (regions II and III).

Despite the observation that all known L. monocytogenes flagellar motility genes were subject to MogR regulation, the microarray analyses did not distinguish if MogR directly or indirectly regulated a given gene. To determine which genes were direct targets of MogR repression, and to potentially define a MogR-recognition site, gel shift analyses were performed with the putative promoter regions of several flagellar motility genes. Candidate promoter regions were chosen based upon the presence of an extended intergenic region or prior identification of a transcriptional start site [8,14,15]. Since our previous studies indicated that MogR present in L. monocytogenes extracts could bind to DNA comprising the *flaA* promoter region [8], we examined whether purified His_6_-tagged MogR could bind to the *flaA* promoter region. Importantly, addition of a His_6_-tag to the C terminus of MogR did not affect repression of *flaA* transcription at 37 °C in L. monocytogenes (unpublished data). When radiolabeled DNA comprising the *flaA* promoter was incubated with increasing amounts of His_6_-tagged MogR, shifted, supershifted, and super-supershifted DNA complexes were observed (Figure 3A). MogR binding to the *flaA* promoter region was specific, as addition of excess unlabeled *flaA* promoter region probe, but not *hly* promoter region DNA, competed away MogR binding to the radiolabeled *flaA* promoter region DNA (Figure 3A).

We next determined whether purified MogR was sufficient to bind the upstream regions of additional MogR-regulated flagellar motility genes (Table 2). Whereas MogR binding to the *motA* and *lmo0693* upstream regions could not be detected (unpublished data), MogR was able to bind and shift radiolabeled DNA probes comprising the *lmo0675, cheY, lmo0703, lmo0723,* and *lmo1699* promoter regions (Figure 3B and unpublished data). Subsequent primer extension analyses detected transcription start sites for the five latter genes, whereas no transcriptional start site was detected for *motA* or *lmo0693* (unpublished data). Thus, these results suggest that MogR represses transcription of *lmo0675, cheY, lmo0703, lmo0723,* and *lmo1699* by binding directly to their promoter regions. Interestingly, supershifted DNA complexes were detected for all five DNA probes, suggesting that multiple MogR binding sites were present within each promoter region or that MogR binding to the DNA induced further multimerization of MogR protein. To assess whether multiple MogR binding sites were present within MogR target promoter regions, DNase I footprinting analysis was performed using *flaA* promoter region DNA. These studies revealed that MogR bound to three regions within the *flaA* promoter DNA (Figure 3C). At low MogR concentrations, MogR protected DNA comprising the *flaA* −35 promoter element (region I), while at higher MogR concentrations, MogR protected regions proximal (region II) and distal (region III) to the −35 element (Figure 3C and 3D). Gel shift analyses with truncated fragments of the *flaA* promoter region confirmed the presence of the three discrete MogR binding regions within the *flaA* promoter region DNA (Figure S1). Taken together, these results suggest that the supershifted complexes detected by gel shift analyses (Figure 3A and 3B) represent binding of MogR to multiple recognition sites within each promoter region.

### MogR Binds Multiple TTTT-N_5_-AAAA Recognition Sites within the Promoter Regions of Flagellar Motility Genes

**Figure 4:**
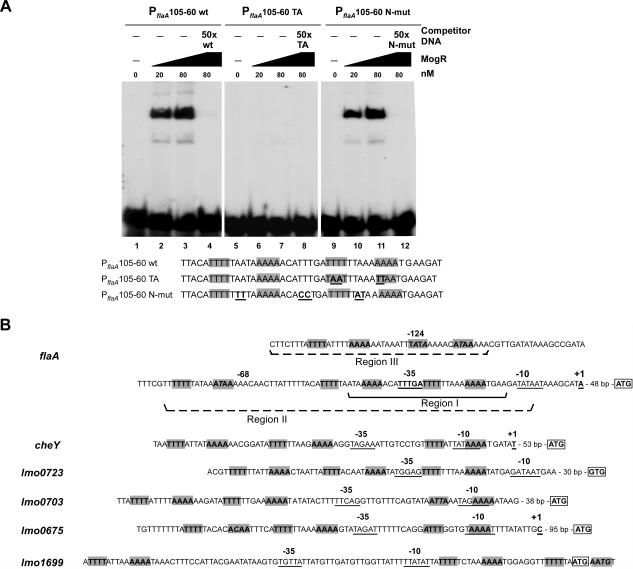
MogR Recognizes a Minimum of Two TTTT-N_5_-AAAA Sites (A) Gel shift analysis of MogR binding to the −35 region of the *flaA* promoter. Radiolabeled *flaA* probe DNA spanning region −58 to −13 relative to the transcription start site was incubated with increasing concentrations of purified His_6_-tagged MogR (lanes 2 to 4, 6 to 8, and 10 to 12) and in the presence of unlabeled competitor DNA (lanes 4, 8, and 12). Radiolabeled wild-type (wt) probe was used in lanes 1 to 4; radiolabeled probe harboring mutations in four residues of one binding site (TA) was used in lanes 5 to 8; and a radiolabeled probe harboring mutations in six residues interspersed between the predicted MogR contact sites (N-mut) was used in lanes 9 to 12. Sequences of the DNA probes are given below the panel. Mutations introduced are underlined and in bold. Gray boxes denote putative MogR binding sites. The binding reactions were analyzed by nondenaturing PAGE. The identity of unlabeled competitor DNA added in 50-fold excess to binding reactions is indicated. (B) Sequences of predicted MogR binding sites within the promoter regions of genes directly regulated by MogR. Gray boxes around text in bold indicates predicted MogR binding sites with italicized text indicating deviations from the consensus TTTT-N_5_-AAAA binding site. The transcriptional start sites of *flaA, cheY,* and *lmo0675* have been precisely mapped by primer extension and are designated by +1. The approximate transcriptional start regions for *lmo0723, lmo0703,* and *lmo1699* have been identified by primer extension. The predicted −35 and −10 elements are underlined*.* The distance to the coding region is indicated, and the translation initiation codon is boxed and in bold. Region I, and regions II and III correspond to the high and low affinity binding sites, respectively, mapped by DNase I footprinting. Region I is denoted by a solid bracket. Regions II and III are marked by a dashed bracket.

Microarray analyses followed by subsequent gel shift assays identified six promoter regions subject to direct regulation through MogR binding. We used these promoter region sequences as a training set for the MEME motif discovery tool to define a consensus-binding site for MogR. The most significant ungapped motif that occurred multiple times within the input promoter regions was TTTT-N_5_-AAAA. Significantly, this sequence appeared twice within the −35 region of the *flaA* promoter and corresponded to the high affinity MogR-binding site mapped by DNase I footprinting (Figure 3D, region I). To test whether MogR specifically recognized TTTT-N_5_-AAAA sites, MogR binding to a 45-bp radiolabeled DNA probe comprising the −35 element of the *flaA* promoter was examined by gel shift analysis. MogR bound and shifted the 45-bp DNA probe, while addition of unlabeled excess competitor DNA abrogated MogR binding (Figure 4A, wt). Mutation of four residues within one of the TTTT-N_5_-AAAA sites was sufficient to prevent MogR binding (Figure 4A, TA), whereas mutation of residues outside the predicted recognition sites did not affect MogR binding (Figure 4A, N-mut). Taken together, these results suggest that a minimum of two TTTT-N_5_-AAAA sites constitutes a MogR recognition site.

Analysis of the distribution of TTTT-N_5_-AAAA sequences within the known MogR-bound promoter regions revealed the presence of multiple sites within each promoter region. A putative TTTT-N_5_-AAAA MogR binding site either overlapped a portion of the −35 or −10 promoter element of each MogR target gene, or the recognition sequence was immediately adjacent to the −35 or −10 promoter element, implying that MogR directly represses transcription by preventing RNA polymerase (RNAP) from binding to these promoter elements (Figure 4B). Furthermore, the level of DNase I protection of *flaA* promoter regions by MogR correlated with the extent of deviation from the TTTT-N_5_-AAAA consensus sequence (Figure 3C). Region I contains two perfect consensus recognition sites (Figure 3C and 4B); region II contains a recognition site with one mismatch; and region III, which MogR bound with the lowest affinity, contains two recognition sites with a total of three mismatches (Figure 4B). In contrast, consensus MogR binding sites were not found within the upstream regions of *motA* and *lmo0693,* which MogR failed to bind in gel shift analyses (unpublished data). However, transcriptional start sites for these genes have not been determined.

### Use of the TTTT-N_5_-AAAA Recognition Motif to Identify MogR-Regulated Genes

To determine which genes identified by microarray analysis might be directly regulated by MogR, we conducted a genome-wide search for TTTT-N_5_-AAAA sequences in the upstream regions of L. monocytogenes coding sequences (300 bp upstream). A total of 4884 TTTT-N_5_-AAAA sites (one mismatch allowed) were identified within the L. monocytogenes upstream regions. Since the promoter regions of the six known MogR-target genes contained a minimum of two TTTT-N_5_-AAAA sites typically separated by seven to nine or 17 to 19 base pairs (equivalent to one or two helix turns, respectively), we restricted our search to upstream regions containing at least two binding sites (one mismatch allowed) spaced one or two helix turns apart. Twenty-five genes were identified as candidates for direct MogR regulation, including five of the six known MogR-target promoters (Table S2). Although *lmo1699* was not identified by these criteria, one of its MogR binding sites is found within the coding sequence (Figure 4B). Therefore, these six promoter regions likely control expression of all known flagellar motility genes based on the occurrence of transcriptional terminators within this region of the chromosome (Table 2).

Only one other gene identified by the restricted motif search was identified as MogR-regulated by microarray analysis (Table S2, >3.5-fold change, *p* < 0.01). Two MogR binding sites were found in the promoter region of *lmo1266,* a gene of unknown function that was repressed 11-fold by MogR during growth in BHI broth at RT (Table S2). None of the remaining 19 candidate genes had statistically significant changes in gene expression observed by microarray analysis. This may indicate that MogR regulates expression of these genes under different growth conditions or that additional factors govern constitutive expression of these genes under the growth conditions used in our microarray analysis. Alternatively, these upstream regions may not contain a functional promoter. To determine if MogR could bind additional identified recognition sites, MogR binding to the upstream regions of two candidate genes, *gbuA* and *lmo2165,* was examined by gel shift analysis. MogR bound and induced supershifted species for both upstream regions (Figure S2), indicating that two TTTT-N_5_-AAAA binding sites spaced one or two helix turns apart can predict binding targets of MogR. Although the precise spacing between recognition sequences required for MogR binding is unknown**,** the requirement for multiple binding sites likely confers specificity to MogR recognition in the AT-rich genome of L. monocytogenes [16]. Interestingly, none of the genes identified as being MogR-activated were recognized by the restricted motif search as genes potentially regulated by MogR directly. Thus, MogR-activated genes may be directly regulated by MogR through recognition of an alternate binding site or indirectly regulated by MogR via a secondary effector.

### MogR Protein Levels in L. monocytogenes Are Temperature Independent

**Figure 5:**
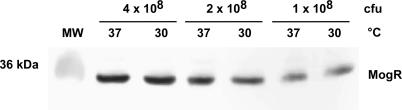
MogR Protein Levels Are Temperature Independent Western blot analysis of MogR protein levels from wild-type L. monocytogenes. Whole cell lysates prepared from 14- to 16-h cultures grown at 37 °C or 30 °C were separated on a 12% SDS-PAGE gel and analyzed by Western blot using a MogR-specific antibody. Number of CFUs of bacteria analyzed and growth temperature is indicated.

DNA binding analyses suggested that MogR mediates transcriptional repression of flagellar motility genes by preventing RNAP from binding to these gene promoters. However, the mechanism controlling stringent MogR repression of flagellar motility gene expression at elevated temperature remained unclear. To determine whether temperature regulation was mediated by an increase in MogR protein levels at elevated temperatures, we performed Western blot analysis of wild-type L. monocytogenes grown at different temperatures. No difference in MogR protein levels was detected during growth in BHI broth at 37 °C, 30 °C (Figure 5), or RT (unpublished data). Growth phase−regulated expression of MogR was also not observed (unpublished data). Thus, MogR repression activity, rather than MogR protein levels, is temperature regulated.

### The DegU Response Regulator Is Required for Modulation of MogR Repression Activity and Regulates FlaA Production at a Posttranscriptional Level

Three possible mechanisms can be envisioned to explain the temperature-dependent regulation of *flaA* transcription. First, MogR repression activity may be temperature regulated by its inherent structure; second, a transcriptional activator may compete with MogR for binding to the *flaA* promoter region specifically at low temperatures; or third, a trans-acting factor may antagonize the repression activity of MogR to permit transcription at low temperatures. No difference in MogR binding to *flaA* promoter region DNA was observed when gel shift analyses were performed at different temperatures using purified MogR protein (unpublished data). Thus, an additional *trans*-acting factor likely regulates MogR repression function in a temperature-dependent manner.

**Figure 6:**
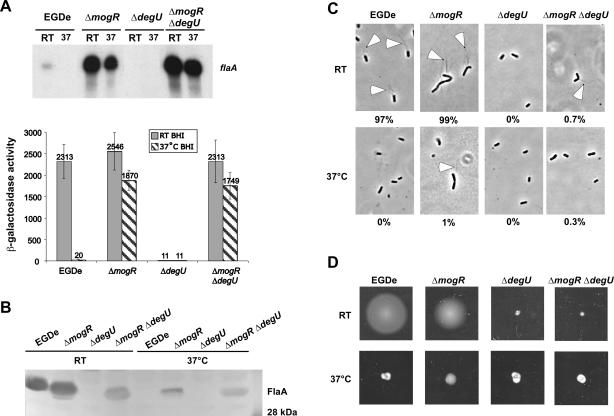
DegU Is Required to Alleviate MogR Repression Activity and for Posttranscriptional Regulation of FlaA Production (A) Analysis of *flaA* transcript levels. L. monocytogenes strains wild-type, Δ*mogR,* Δ*degU,* and Δ*mogR ΔdegU* were grown 14 to 16 h in BHI broth at RT or 37 °C. RNA was harvested and *flaA* transcript levels were analyzed by primer extension. *flaA* promoter activity was also determined by β-galactosidase assays using *flaA*::Tn917 transposon insertion derived strains grown similarly at RT or 37 °C. β-Galactosidase activities represent the means and standard deviations of three independent experiments. (B) Western blot analysis of FlaA protein levels in the strains used in (A). Whole cell lysates were prepared from bacterial cultures described in (A), separated on a 12% SDS-PAGE gel, and analyzed by Western blot using a FlaA-specific antibody. (C) Flagellar staining of strains described in (A). Cultures grown at 37 °C or RT were stained for flagella and analyzed by microscopy. Open arrowheads indicate flagella. The percentage of bacteria harboring at least one flagellum is given below. (D) Motility analysis of strains used in (A). A single colony was inoculated with a straight needle in low-agar (0.375%) BHI plates and incubated at 37 °C or RT for 48 h.

The DegU response regulator has recently been shown to be required for transcription of *flaA*, as deletion of *degU* ablates *flaA* expression even at low temperatures [9,17]. Consequently, it has been proposed that DegU functions as a transcriptional activator of the *flaA* promoter [9]. To test this hypothesis, we examined the effect on *flaA* transcription of deleting *degU* in the Δ*mogR* background (Δ*mogR ΔdegU,* DH-L1274). In Δ*mogR*, transcription of *flaA* is de-repressed at both low and elevated growth temperatures (Figure 6A). However, if DegU is required to activate *flaA* transcription, Δ*mogR ΔdegU* bacteria should contain lower levels of *flaA* transcripts relative to Δ*mogR*. No significant difference in *flaA* transcription was detected between Δ*mogR ΔdegU* and Δ*mogR* when *flaA* transcript levels were determined by primer extension and *flaA* expression was analyzed using a *flaA* promoter-*lacZ* transcriptional fusion (Figure 6A). This result demonstrates that DegU is not required to activate *flaA* transcription and strongly suggests that DegU is required to relieve MogR-mediated repression of *flaA* at low temperatures.

**Figure 7:**
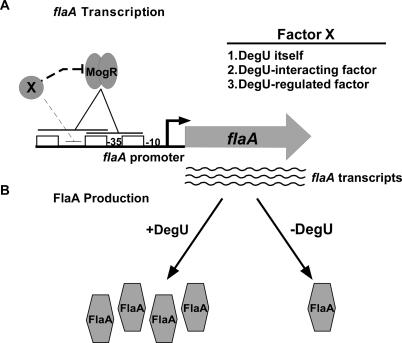
Model for the Regulation of FlaA Expression at Low Temperatures in L. monocytogenes (A) Binding of MogR to TTTT-N_5_-AAAA sites (open boxes) in the *flaA* promoter region represses *flaA* transcription by occluding RNAP binding (solid lines over open boxes). The *flaA* coding region is represented as a filled block arrow; transcription initiating from the *flaA* promoter is represented as a bent arrow; and the −35 and −10 sites are marked. At low temperatures, MogR repression activity is antagonized by an unknown factor *(X).* This factor may be DegU, a DegU-interacting factor, and/or a DegU-regulated factor. *X* may modulate MogR repression activity by either interacting directly with MogR (thick dashed line) or binding to the *flaA* promoter region (thin dashed line) impeding MogR binding to TTTT-N_5_-AAAA sites. (B) DegU regulates FlaA protein levels through a posttranscriptional mechanism. At low temperatures when *flaA* transcripts are present (wavy lines), DegU functions to maximize FlaA protein production, either by enhancing translation of *flaA* transcripts or stabilizing FlaA protein.

Interestingly, even though *flaA* was transcribed at similar levels in both Δ*mogR ΔdegU* and Δ*mogR* bacteria (Figure 6A), FlaA protein levels were greatly reduced in Δ*mogR ΔdegU* relative to Δ*mogR* in whole cell lysates (Figure 6B). Analysis of bacterial cytoplasmic and surface-extracted fractions yielded similar results (unpublished data). The reduced level of flagellin correlated with a significant reduction in the number of flagellated Δ*mogR ΔdegU* bacteria detected at RT compared to Δ*mogR* bacteria (0.7% versus 99%, respectively) (Figure 6C) and the loss of motility on low agar plates (Figure 6D). These results indicate that, in addition to modulating the ability of MogR to repress flagellar motility genes at low temperatures, DegU functions to regulate FlaA production at a posttranscriptional level, perhaps by enhancing translation of *flaA* transcripts or stabilizing FlaA protein (Figure 7).

## Discussion

Although temperature-dependent expression of flagellar motility genes has long been established in *L. monocytogenes,* the mechanisms conferring temperature specificity have remained elusive. We previously demonstrated that MogR is a transcriptional repressor of flagellar motility genes and is also required for virulence. In this report, we determined the specific requirement of MogR for virulence of L. monocytogenes and the mechanism by which MogR mediates transcriptional repression.

### The Role of MogR During In Vitro and In Vivo Infection

During infection of host cells, transcriptional profiling revealed that MogR functions primarily to repress flagellar motility gene expression (Table 2). This result suggested that overexpression of flagellar motility genes was largely responsible for the attenuated virulence of MogR-negative bacteria. Overexpression of FlaA was particularly deleterious to infection, as deletion of *flaA* in a MogR-negative background largely restored virulence to MogR-negative bacteria (Table 1). Hyperproduction of FlaA by MogR-negative bacteria during growth at RT induced a chaining phenotype (Figure 1A and 1B). The temperature-dependent chaining effect subsequently impaired host cell invasion by MogR-negative bacteria and likely accounts for the 25-fold more severe virulence defect observed in MogR-negative bacteria when grown at RT rather than at 37 °C prior to infection (Figure 1B and Table 1). While it remains possible that FlaA produced by MogR-negative bacteria inappropriately activates TLR5 signaling [2], this hypothesis seems less likely given that MogR-negative bacteria cultured at 37 °C prior to infection, a condition where FlaA levels are dramatically reduced, still exhibited a 10-fold virulence defect and an approximately 20% intracellular spreading (plaquing) defect (Table 1). Furthermore, deletion of *flaA* failed to fully alleviate the plaquing and virulence defect of MogR-negative bacteria (Table 1). These defects may result from altered localization of proteins required for intracellular motility, or insufficient energy being available for production of other virulence factors due to overexpression of flagellar motility genes in MogR-, FlaA-negative bacteria.

### MogR Is a Transcriptional Repressor of Flagellar Motility Gene Expression

Additional microarray analysis revealed that MogR mainly represses flagellar motility gene expression during growth at 37 °C in BHI broth, as far fewer flagellar motility genes were MogR repressed during growth in BHI at RT (Table 2). Genes identified as MogR repressed (minimum 3.5-fold change, *p* < 0.01) were more strongly repressed during growth at 37 °C (27-fold) than at RT (5-fold). Furthermore, MogR activated expression of more genes (96) than it repressed (54) during growth at RT (Figure 2A, Table S1). Many of the MogR-activated genes encode products related to sugar transport and metabolism that may function to supply energy for flagella production or provide metabolic inputs regulating flagella production [18]. Thus, microarray analyses revealed that MogR repression activity was temperature regulated.

Gel shift analyses revealed that purified MogR specifically bound to and induced supershifted complexes with six flagellar motility gene promoter regions, suggesting that multiple MogR binding sites were present within each promoter region. Indeed, additional DNA binding analyses identified three discrete MogR binding sites within *flaA* promoter region DNA (Figures 3C and S1). MEME motif analyses revealed that the putative MogR binding site, TTTT-N_5_-AAAA, occurred multiple times within each known MogR target promoter region. Given that putative MogR recognition sequences flanked either the −35 or −10 elements, MogR likely represses flagellar motility gene expression by occluding RNAP binding (Figure 4B). Consistent with this hypothesis, two perfect TTTT-N_5_-AAAA sites straddle the −35 element of the *flaA* promoter and are sufficient to mediate MogR binding in vitro (Figure 4A and 4B). Mutation of four residues within one of these binding sites abrogated MogR binding to the *flaA* promoter region by gel shift analysis (Figure 4A), confirming that MogR requires at least two TTTT-N_5_-AAAA sequences to bind DNA.

We also observed that MogR binding sites were spaced one or two helix turns apart within the flagellar gene promoter regions (Figure 4B). When a genome-wide search for TTTT-N_5_-AAAA sequences spaced one or two helical turns apart was performed, 25 genes were identified as potential targets of direct MogR regulation. However, only six of the 25 candidate MogR target genes were found to be MogR regulated under the conditions used for microarray analysis (Table S2), of which five were flagellar motility genes and known MogR targets. The remaining MogR-regulated gene, *lmo1266,* is a gene of unknown function that is currently under investigation. Although it is unclear whether the upstream regions of the other nonflagellar motility candidate genes contain a functional promoter, MogR binding in vitro to upstream regions of two candidate genes *(gbuA* and *lmo2165)* containing two TTTT-N_5_-AAAA sites could be detected (Figure S2). Although the specific spacing and/or topological requirements for MogR binding remain to be defined, these results indicate that at least two TTTT-N_5_-AAAA sites spaced one or two helix turns apart are required for MogR binding.

Interestingly, the TTTT-N_5_-AAAA MogR binding site is the reverse of the Bacillus subtilis ComK binding site, which requires that ComK sites be spaced one, two, or three helical turns apart [19]. This observation may explain why the competence-related gene, *comC,* was identified as being MogR-activated during growth at physiological temperature (Table S1). Although two AAAA-N_5_-TTTT sequences (one or two mismatches) are present in the *comC* upstream region, MogR failed to appreciably bind this upstream region by gel shift analysis (unpublished data), perhaps due to inappropriate spacing of these sites. At present, it remains to be determined whether MogR functions as a transcriptional activator.

### Temperature-Dependent Regulation of MogR Function

While our studies suggested a mechanism by which MogR directly acts as a transcriptional repressor, they did not reveal why MogR only partially represses flagellar motility genes at RT. MogR protein levels were found to be temperature independent (Figure 5), suggesting that a *trans*-acting factor may regulate MogR repression function. One candidate factor was the DegU response regulator, which is required for *flaA* transcription at low temperatures [9]. However, epistatic analyses indicated that DegU was dispensable for *flaA* transcription in the absence of MogR (Figure 6A), demonstrating that DegU does not function as a transcriptional activator for *flaA*. Recent studies have also implicated a role for DegU in permitting expression of all known flagellar motility genes at low temperatures [20], a result that we have independently confirmed. Our preliminary studies also indicate that flagellar motility genes are similarly expressed in both MogR-, DegU-negative and MogR-negative bacteria at RT (unpublished data). These results collectively imply that DegU directly or indirectly antagonizes MogR repression function at multiple promoters at low temperatures.

Although DegU is clearly required to permit flagellar gene expression in the presence of MogR at low temperatures, the mechanism by which DegU modulates MogR repression activity remains unclear. DegU itself, or DegU in concert with an unidentified factor, may interfere with MogR repression function. Alternatively, DegU may activate transcription of an unidentified factor that antagonizes MogR function. The proposed factor(s) may be either a protein or metabolite co-factor that modulates MogR activity by either binding to the *flaA* promoter region or sequestering MogR from its target sites. Although the proposed scenarios for modulation of MogR activity are not mutually exclusive, they all must involve DegU (Figure 7). Transcription of *degU* is not temperature dependent [20], and DegU protein is detectable at both elevated and low temperatures (unpublished data), so changes in DegU activity likely mediate temperature specificity. As a response regulator, phosphorylation of L. monocytogenes DegU may control its activity, given that the highly homologous B. subtilis DegU is regulated by phosphorylation by its cognate sensor kinase, DegS [21]. A cognate DegS sensor kinase, however, is conspicuously absent from the genome of L. monocytogenes.

In addition to its role in regulating *flaA* transcription, DegU was found to regulate flagella production through a posttranscriptional mechanism. Despite producing equivalent levels of *flaA* transcripts, MogR-, DegU-negative bacteria produced markedly lower FlaA protein than both wild-type and MogR-negative bacteria during growth at low temperatures (Figure 6B). We are currently investigating whether changes in FlaA stability mediate the decrease in FlaA levels in MogR-, DegU-negative bacteria.

### Nonhierarchal Expression of Flagellar Motility Genes in L. monocytogenes

Flagellar production is an energy-intensive process that is subject to multiple levels of regulation [18]. In almost all bacteria studied to date, a master transcriptional activator induces expression of flagellar motility genes in a hierarchical manner to allow ordered and prudent expression of flagellar components [18,22]. An alternative sigma factor, FliA, activates expression of the last temporal class of flagellar motility genes, which includes genes for flagellins. However, expression of FliA-regulated genes is contingent upon completion of the hook-basal body, which allows export of the anti-sigma factor FlgM from the cytoplasm [23,24]. In contrast, a transcriptional repressor uniformly downregulates flagellar motility gene expression in L. monocytogenes. In the absence of MogR, all flagellar motility genes are constitutively transcribed at elevated levels, and no transcriptional activator of these genes has been identified. FliA and FlgM homologs are also absent from the genomes of *Listeria* species, and *flaA* expression does not require completion of the hook-basal body structure in L. monocytogenes [25]. Thus, expression of flagellar motility genes appears to occur in a nonhierarchical manner in L. monocytogenes. Interestingly, expression of flagellin is similarly independent of hook-basal body formation in Bacillus thuringiensis [26]*,* a member of the B. cereus group that is genetically indistinguishable from Bacillus cereus and Bacillus anthracis [27,28]. The B. cereus group also lacks FliA and FlgM homologs [26], and it is the only other set of organisms known to encode MogR homologs [8]. Thus, it is tempting to speculate that the B. cereus group and *Listeria* species share a novel regulatory mechanism for flagella production whereby a transcriptional repressor, MogR, functions as the master regulator to control nonhierarchical expression of flagellar motility genes.

## Materials and Methods

### Bacterial and eukaryotic cell growth conditions.

L. monocytogenes and E. coli strains used in this study are listed in Table S3. *L. monocytogenes* strains were grown at temperatures of 30 °C, 37 °C, or RT (18 °C to 25 °C) in BHI medium (Difco, BD Biosciences, San Diego, California, United States). E. coli strains were grown in Luria-Bertani (LB; Difco) medium at 37 °C with shaking. The mouse cell lines J774 and L2 were maintained at 37 °C in a 5% CO_2_-air atmosphere [29].

### Strain and plasmid construction.

Antibiotics were used at the following concentrations: chloramphenicol 20 μg/ml for selection of pPL2 derivatives in E. coli and 7.5 μg/ml for selection of integrated pPL2 derivatives in L. monocytogenes; 100 μg/ml carbenicillin for pCON1 derivatives, and 30 μg/ml kanamycin for pET29b vectors in E. coli; 1 μg/ml erythromycin for selection of L. monocytogenes strains with Tn917-derived transposon insertions. Strains are listed in Table S3, and primers are listed in Table S4. Strain Δ*mogR ΔflaA* (DH-L1248), containing an in-frame deletion in the *flaA* gene in Δ*mogR,* was created by allelic exchange [30] using plasmid pKSV7Δ*flaA* [31]. To construct an in-frame deletion of *degU,* primers 464 and 465 were used with wild-type EGDe genomic DNA to amplify approximately 0.8 kb of the region upstream of *degU*. Primers 466 and 467 and EGDe genomic DNA were used to amplify approximately 1 kb of sequence downstream of *degU*. The 5′ and 3′ PCR products were gel purified using the QIAquick gel extraction kit (Qiagen, Valencia, California, United States) and used as templates for a splicing by overlap extension PCR [32]. The flanking primers, 464 and 466, were used to amplify an approximately 1.8-kb PCR product containing an in-frame deletion of amino acids 17 to 216 of *degU.* The splicing by overlap extension PCR product was gel purified, digested with XbaI and KpnI, ligated to plasmid pCON1 digested with the same restriction enzymes, and transformed into XL1-Blue to create strain DH-E1272. The resulting plasmid, pCON1/Δ*degU*, was sequenced and introduced into wild-type and Δ*mogR* by electroporation, and allelic exchange was performed to generate strains Δ*degU* (DH-L1273) and Δ*mogR* Δ *degU* (DH-L1274), respectively. A similar procedure was used to delete *degU* from *flaA*::Tn917–containing strains, DH-L975 and DH-L1179, to generate strains DH-L1275 and DH-L1276, respectively.

### Determination of LD_50_ values.

LD_50_ values were determined as previously described [33]. All animal experiments were conducted with approval from the institutional IACUC.

### Invasion assays.

Six-well dishes were seeded with 2 × 10^6^ L2 cells in RPMI supplemented with 10% fetal bovine serum (HyClone, Logan, Utah, United States) and 2 mM glutamine (Mediatech, Herndon, Virginia, United States). Each well contained four sterile 12-mm-diameter glass coverslips. Cells were incubated for 24 h until a confluent monolayer formed. Fourteen- to 16-h cultures of L. monocytogenes strains grown at 37 °C or RT were washed once with PBS and used to infect L2 cells at an MOI of 30. The infection was allowed to proceed for 1 h at 37 °C, after which infected monolayers were washed three times with PBS, and RPMI medium containing 50 μg/ml gentamicin was added to kill extracellular bacteria. Thirty minutes later, coverslips were removed, host cells were lysed, and intracellular colony forming units (CFUs) were determined. The number of CFUs in the initial inoculum was also determined.

### Plaque formation assay in L2 fibroblasts**.**

Plaquing assays were performed as described [8], except that cultures were grown at either RT or 37 °C prior to infection.

### Western blot analysis of FlaA.

For detection of FlaA in cellular fractions, 8-ml cultures of L. monocytogenes were grown at RT lying flat for 20 h and centrifuged at 7,000*g* for 10 min. The resulting supernatant was precipitated in 10% TCA (final concentration) for 1 h on ice. Precipitates were pelleted at 7,000g for 10 min, and the resulting pellet was resuspended in 200 μl of 1× final sample buffer (FSB) containing 0.1 N NaOH. Cytoplasmic and surface-extracted fractions were prepared as described [8]. A culture volume equivalent to 1.2, 0.4, and 2 ml for supernatant, surface-extracted, and cytoplasmic fractions, respectively, was resolved on a 12% SDS-PAGE gel. Western blot analysis was performed as described [8], except that goat anti-rabbit horseradish peroxidase–conjugated secondary antibody (BioRad, Hercules, California, United States) and Western Lightning (Perkin Elmer, Wellesley, Massachusetts, United States) ECL detection method were used. For whole cell lysate samples, 1 ml of L. monocytogenes cultures grown in BHI medium for approximately 24 h at RT and 37 °C was pelleted, resuspended in 100 μl of TE/lysozyme (10 mM Tris-HCl [pH 8.0], 1 mM EDTA, 2 mg/ml lysozyme), and incubated at 37 °C for 1 h. Next, 100 μl of 2× FSB was added, samples were boiled for 5 min at 95 °C and then centrifuged for 5 min at 16,000*g*, and a culture volume equivalent to 0.2 ml of an OD_600_ = 1.1 was loaded onto a 12% SDS-PAGE gel. Western blot analysis was performed as described [8] using a rabbit anti-*Listeria* FlaA antibody (Denka Seike, Tokyo, Japan).

### Microscopic analysis.

L. monocytogenes was grown 14 to 16 h at RT or 37 °C in 2 ml of BHI medium without shaking. Flagella were detected using a crystal violet stain and analyzed by light microscopy [34]. The percentage of bacteria that possessed at least one flagellum was derived from inspecting 300 individual bacteria per culture. A chaining event was defined as a chain of three or more bacteria. A total of 300 bacterial events were counted for each population.

### Microarray analyses.

RNA from bacteria growing in BHI broth cultures was isolated as previously described [8]. RNA from bacteria growing intracellularly was isolated as previously described [35], with the exception that RNA was harvested 9 h postinfection. Total RNA 10 μg was mixed with 2 μg of random hexamer DNA (Invitrogen, Carlsbad, California, United States) in a total volume of 9.5 μl. Fluorescently labeled cDNA was prepared by direct incorporation of fluorescent nucleotide analogs (Cy3-dCTP and Cy5-dCTP) during a first-strand, randomly primed reverse transcription reaction using Superscript III reverse transcriptase according to the manufacturer's instructions (Invitrogen). A modified mix of dNTPs was used (0.2 mM dG/T/ATP, 0.1 mM dCTP) with 1 μl of Cy3-dCTP or Cy5-dCTP (GE Biosciences, Piscataway, New Jersey, United States). Following reverse transcription, the RNA was degraded by heating samples at 95 °C for 3 min, adding 1 μl of 2 N NaOH, and incubating samples at 37 °C for 10 min. Samples were neutralized by adding 1 μl of 2 N HCl, 5 μl of 1 M Tris-HCl (pH 7.5), and 23 μl of distilled water. Extension products were purified using the QIAquick PCR purification kit (Qiagen) according to the manufacturer's instructions, except that samples were eluted in 2× 100 μl of elution buffer. DNA was precipitated by adding 2.5 μl of 1 mg/ml salmon sperm DNA, 40 μl of 3 M sodium acetate, and 720 μl of 100% ethanol. Labeled DNA was recovered by centrifugation, washed once in 70% ethanol, air-dried for 3 min at RT, and then resuspended in 30 μl hybridization buffer (25% formamide, 5× SSC, 1% SDS) by gentle vortexing, followed by heating at 95°C for 5 min, then pipeting to resuspend, followed by heating at 95 °C for a second time. The differentially labeled samples were then mixed together and competitively hybridized to L. monocytogenes microarrays (TIGR, Rockville, Maryland, United States) that had been UV cross-linked and prehybridized at 42 °C in 5× SSC, 0.1% SDS, 10 mg/ml BSA for a minimum of 1 h, washed three times in dH_2_O, and then immersed in 95% EtOH and dried by centrifugation. Hybridization was carried out under Lifterslips (Erie Scientific, Portsmouth, New Hampshire, United States) in a sealed wet box at 42 °C for 14 to 16 h. Following hybridization, slides were washed for 10 min in each of the following solutions: once in 2× SSC, 0.1% SDS at 42 °C; once in 0.2× SSC, 0.1% SDS at RT; twice in 0.2× SSC, and once in dH_2_O at RT, and then dried by centrifugation. Microarray slides were scanned by using a ScanArray 5000 apparatus (GSI Lumonics, Billerica, Massachusetts, United States) and analyzed with GenePix PRO 3.0 software (Axon Instruments, Molecular Devices, Sunnyvale, California, United States). Arrays were repeated with a minimum of two biological samples subjected to fluor reversal and uploaded onto the Rosetta Resolver microarray analysis platform (Rosetta Biosoftware, Seattle, Washington, United States). Individual arrays were weighted by error and combined. Genes were determined to be differentially regulated in MogR-negative bacteria if the absolute fold change was greater than 3.5 with a *p*-value of <0.01. Microarray results were confirmed by primer extension analyses for *lmo0675, lmo0703, lmo1699, lmo0723, flaA,* and *cheY;* Northern blot analysis for *lmo0673, lmo0675, lmo0703, lmo0710, lmo0697, flaA,* and *cheY;* and RT-PCR for *lmo0688, flaA, cheY,* and *lmo0710*.

### Purification of His-tagged MogR.

Primer pair 462 and 463 was used to amplify the *mogR* coding sequence without the stop codon using EGDe genomic DNA as the template. The resulting PCR product was digested with NdeI and XhoI and ligated into the pET29b expression vector digested with the same restriction enzymes. The resulting ligation was transformed into XL1-Blue to create strain DH-E1334. The pET29b/*mogR* plasmid was isolated from DH-E1334, sequenced, and transformed into BL21(DE3) (Novagen, EMD Biosciences, San Diego, California, United States) to create strain DH-E1335. DH-E1335 was grown in LB medium at 37 °C to mid-exponential phase, and expression of MogR was induced with 1 mM IPTG (isopropyl-β-thiogalactoside). The induced culture was grown at 30 °C for 4 h. Bacteria were pelleted, and His_6_-tagged MogR was purified using Ni-NTA Spin Columns (Qiagen) according to the manufacturer's instructions. Purified His_6_-tagged MogR was dialyzed overnight in 1× binding buffer (BB) (100 mM NaCl, 10% glycerol, 1 mM MgCl_2_, 10 mM Tris [pH 7.5], 0.5 mM EDTA, 0.5 mM DTT, 12.5 μg/ml salmon sperm DNA, 50 μg/ml BSA). Protein concentration was determined using a Bradford assay (Sigma, St. Louis, Missouri, United States).

### Gel shift analyses.

DNA probes used for gel shift analyses were radiolabeled by incubating 2.5 pmol of DNA probe with 2 μl of [γ^32^P]ATP (3,000 Ci/mmol; Perkin Elmer) and 10 units of T4 polynucleotide kinase in a total volume of 25 μl. Following incubation at 37 °C for 30 min and heat inactivation at 70 °C for 10 min, 25 μl of TE (10 mM Tris [pH 8.0], 1 mM EDTA) was added, and free ATP was removed by passing the reaction mixture over Sepharose 50 columns (Roche Applied Sciences, Indianapolis, Indiana, United States). For each binding reaction, 0.1 pmol of DNA probe was incubated with a given amount of His_6_-tagged MogR protein in 1× BB for 30 min at 30 °C. Binding reactions were loaded onto a 5% nondenaturing PAGE gel pre-run for a minimum of 2 h at 250 V with 0.05% thioglycolate in 10% glycerol. Reactions were run at 250 V for 2 to 4 h. Primer pairs 489 and 348 *(flaA),* 490 and 376 *(cheY),* 492 and 493 *(lmo0703),* 494 and 495 *(lmo0723),* and 496 and 497 *(lmo1699)* were used for amplification of the DNA probes indicated. For the gel shift mutational analyses, primer pairs 498 and 499, 500 and 501, and 502 and 503 were annealed by heating primer pairs to 70 °C for 10 min in annealing buffer (100 μM Tris-HCl [pH 7.5], 1 M NaCl, 10 mM EDTA), then slow cooling to RT, to yield probes P*_flaA_*105–60 (wt), P*_flaA_*105–60 (TA), and P*_flaA_*105–60 (N-mut).

### DNase I footprinting analyses.

The same *flaA* DNA probe used for gel shift analyses was used for footprinting analyses. To label the noncoding strand of the *flaA* probe, *flaA* DNA probe DNA was digested with XmaI, and 5.0 pmol of probe was labeled with Klenow enzyme and 50 μCi of [α-^32^P]dCTP (3,000 Ci/mmol; Perkin Elmer). Unincorporated nucleotides were removed using Sepharose 50 columns (Roche Applied Biosciences). Binding experiments were performed as for gel shift experiments, except that 0.5 pmol of labeled probe was used. After incubation at 30 °C for 30 min, 1 μl of 10 μg/ml DNase I (Sigma) in 1× BB (supplemented with 10 mM MgCl_2_ and 5 mM CaCl_2_) was added. The digestion reaction was allowed to proceed for 1 min, then 100 μl of 1.2× stop solution (0.6 M Tris [pH 8.0], 12 mM EDTA, 100 μg/ml tRNA) was added to terminate the reaction, and 280 μl of 100% ethanol was added to precipitate the DNA. The resulting DNase I products were resuspended in 5 μl of stop buffer (95% formamide, 20 mM EDTA, 0.05% xylene cyanol, 0.05% bromophenol blue) and analyzed by electrophoresis on a 6% polyacrylamide/8 M urea gel. Maxam-Gilbert G+A reactions were run with each experiment to locate sequence positions and protected regions [36].

### Motif discovery and genome search.

The MEME (http://meme.sdsc.edu) program was used to determine a consensus-binding site motif for MogR. The intergenic sequences comprising 300 bp upstream of the start codon of *flaA, lmo0675, cheY, lmo0703, lmo0723,* and *lmo1699* were input into the MEME program to search for long (gapped) motifs between 14 and 17 bp in width with a minimum of two sites per intergenic region. To search for the occurrence of TTTT-N_5_-AAAA sites within 300 bp of a coding sequence in the genome of *L. monocytogenes,* the “Search Pattern” function on the ListiList server (http://genolist.pasteur.fr/ListiList) was used. For the restricted search, one mismatch was allowed per site and either 7 to 9 or 17 to 19 bp were allowed between sites.

### Western blot analysis of MogR.

One milliliter of L. monocytogenes cultures grown in BHI medium for approximately 24 h at 30 °C and 37 °C was pelleted, resuspended in 100 μl of TE, lysozyme (10 mM Tris-HCl [pH 8.0], 1 mM EDTA, 2 mg/ml lysozyme), incubated at 37 °C for 1 h, and then frozen at −20 °C. A sample of the approximately 24-h culture was also removed for CFU determination. The next day, 100 μl of 2× FSB was added to frozen samples, and samples were boiled for 5 min at 95 °C and centrifuged for 5 min at 16,000*g*, and the equivalent of 4, 2, and 1 × 10^8^ CFUs was loaded onto a 12% PAGE gel. Proteins were visualized by Western blot using a rabbit anti-MogR antibody generated by immunizing a rabbit with affinity-purified His_6_-tagged MogR (Cocalico, Reamstown, Pennsylvania, United States) and a goat anti-rabbit alkaline phosphatase–conjugated secondary antibody (Kierkegaard & Perry Laboratories, Gaithersburg, Maryland, United States).

### Primer extension analyses.

Measurement of *flaA* transcript levels by primer extension analyses was performed as described [8].

### β-Galactosidase measurement of *flaA* promoter activity.

β-Galactosidase assays were performed as described [8], with the exception that samples were diluted 1:10 and 1:20 in AB buffer (60 mM K_2_HPO_4_, 40 mM KH_2_PO_4_, 100 mM NaCl) prior to mixing with MUG substrate.

### Data deposition.

The microarray datasets and Rosetta Resolver analyses reported in this paper have been deposited in the Gene Expression Omnibus database under accession number GSE3655 (http://www.ncbi.nlm.nih.gov/geo/query/acc.cgi?acc = GSE3655).

## Supporting Information

Figure S1 MogR Recognizes Three Discrete Sites within *flaA* Promoter Region DNA (A) Schematic representation of probes used in gel shift analysis of MogR binding to *flaA* promoter region DNA. Solid lines represent the *flaA* promoter region DNA spanned by each probe. The identity of the probes is indicated in parentheses with numbers given relative to the transcriptional start site. Dashed lines demarcate discrete MogR binding sites within the *flaA* promoter region as determined by gel shift analyses.
(B) Gel shift analysis of MogR binding to varying regions of *flaA* promoter DNA. Radiolabeled *flaA* DNA probe spanning regions −162 to +8, −162 to −92, −93 to −13, −124 to −34, and −68 to +8 was incubated with increasing concentrations of purified His_6_-tagged MogR (indicated above each lane). Shifted (S), super-shifted (SS), and super-supershifted (SSS) DNA complexes are indicated. Note that super-shifted species are observed only when a given probe spans at least two predicted MogR binding regions. The relative affinity of MogR for the different probes is (−162 to +8) > (−93 to −13) > (−68 to +8) > (−124 to −34) ≈ (−162 to −92). The affinity of MogR for the *flaA* promoter probes as determined by gel shift analysis corresponds to the affinities observed by DNase I footprinting analysis.
(C) DNA sequence of the −162 to +8 *flaA* promoter probe. Numbers indicate the start and end sites of various *flaA* promoter probes used in (B) and are given relative to the transcriptional start site with the relevant nucleotide underlined. Gray boxes around text in bold indicates predicted MogR binding sites, with italicized text indicating a mismatch from the consensus TTTT-N_5_-AAAA recognition sequence. The transcriptional start site (+1) is indicated in bold, and the −35 and −10 promoter elements are marked with a solid line above the *flaA* sequence. Regions I, II, and III refer to the MogR binding sites defined by DNase I footprinting analyses and are marked by solid brackets.
(1.5 MB TIF)

Figure S2 Purified MogR Is Sufficient to Bind the Upstream Regions of *gbuA* and *lmo2165*
(A) Gel shift analysis of MogR binding to the *gbuA* and *lmo2165* upstream regions. Radiolabeled *gbuA* or *lmo2165* DNA comprising regions −300 to +18 relative to the translational start site were incubated with increasing concentrations of purified His_6_-tagged MogR (lanes 2 to 6) and in the presence of unlabeled competitor DNA (lanes 5 and 6). The identity of the unlabeled competitor DNA added in 25-fold excess to binding reactions is indicated.
(B) DNA sequence of the *gbuA* and *lmo2165* upstream regions. Gray boxes around text in bold indicates predicted MogR binding sites with italicized text indicating a mismatch in the consensus TTTT-N_5_-AAAA sequence. The ATG translational start site is indicated.
(1.5 MB TIF)

Table S1 Microarray Analysis of MogR-Negative L. monocytogenes Relative to Wild-Type during Growth in Different Conditions (87 KB PDF)

Table S2 Putative MogR-Regulated Genes Defined by Genome-Wide Search for TTTT-N_5_-AAAA Sites in Upstream Sequences (56 KB PDF)

Table S3 L. monocytogenes and Escherichia coli Strains (63 KB PDF)

Table S4 Oligonucleotides Used in This Study (38 KB PDF)

### Accession Numbers

The GenBank (http://www.ncbi.nih.gov/Genbank) accession numbers for genes (GeneID) used in this study are *degU* (987300), *flaA* (987167), and *mogR* (985015); the protein accession numbers are DegU (NP_466038), FlaA (NP_464217), and MogR (NP_464201), respectively.

## Abbreviations

BHI: brain heart infusion
FlaA: flagellin
RNAP: RNA polymerase
RT: room temperature
