# Supplementary material for: The MogR Transcriptional Repressor Regulates Nonhierarchal Expression of Flagellar Motility Genes and Virulence in Listeria monocytogenes
Source: PLoS Pathog. 2006 Apr 14;2(4):e30. doi: 10.1371/journal.ppat.0020030 (PMC1435793; doi:10.1371/journal.ppat.0020030)
Supplement: Supplementary file 1 [file ppat.0020030.st001.pdf]

Table S1. Microarray analysis of MogR-negative *L. monocytogenes* relative to wild-type during growth in different conditions.

	37˚C	37˚C	RT	
	J774	BHI	BHI	
lmo0019	*-1.1	**3.7**	3.2	unknown
lmo0036	*2.1	*1.8	**-9.5**	similar to ornithine carbamoyltransferase
lmo0043	*-1.1	2.1	**5.5**	similar to arginine deiminase
lmo0093	*1.2	*-1.3	**4.8**	similar to ATP synthase epsilon chain
lmo0157	*-1.5	**-9.9**	*1.3	similar to ATP dependent helicase
lmo0178	*-1.1	**-3.6**	**-4.9**	similar to xylose repressor
lmo0179	*-1.1	**-4.1**	**-5.0**	similar to sugar ABC transporters, permease proteins
lmo0181	*1.6	**-4.9**	-3.2	similar to sugar ABC transporter, sugar-binding protein
lmo0202	*1.1	*1.8	**-7.3**	listeriolysin O precursor
lmo0254	*1.2	*1.2	**-4.7**	unknown
lmo0262	*-1.5	**4.0**	*1.1	internalin G
lmo0278	*1.1	*-1.3	**-4.4**	similar to sugar ABC transporter, ATP-binding protein
lmo0279	*-1.3	**-4.0**	*-1.2	highly similar to anaerobic ribonucleoside-triphosphate reductase
lmo0280	*-1.1	**-3.8**	-2.1	highly similar to anaerobic ribonucleotide reductase activator protein
lmo0300	*1.3	*-1.5	**-3.8**	similar to phospho-beta-glucosidase and phospho-beta-galactosidase
lmo0321	*1.2	*-1.1	**5.0**	similar to unknown proteins
lmo0322	*1.3	*2.3	**4.0**	similar to unknown proteins
lmo0327	*1.1	*-1.1	**-7.1**	similar to cell surface proteins (LPXTG motif)
lmo0343	*1.4	*-1.5	**-7.8**	similar to transaldolase
lmo0344	*1.3	*-1.3	**-12.1**	similar to dehydrogenase/reductase
lmo0345	*1.1	*-2.0	**-4.1**	similar to sugar-phosphate isomerase
lmo0347	*1.5	*1.1	**-11.2**	similar to dihydroxyacetone kinase
lmo0372	*-1.2	*-1.3	**-4.1**	similar to beta-glucosidase
lmo0377	*1.2	*-1.1	**-3.7**	unknown
lmo0393	*-1.1	-2.2	**-4.1**	unknown
lmo0398	*1.6	**9.9**	**-8.2**	similar to phosphotransferase system enzyme IIA
lmo0399	*1.3	*2.4	**-7.4**	similar to fructose-specific phosphotransferase enzyme IIB
lmo0400	*1.2	**14.5**	**-8.7**	similar to fructose-specific phosphotransferase enzyme IIC
lmo0401	*1.5	*1.8	**-7.0**	highly similar to *E. coli* YbgG protein, a putative sugar hydrolase
lmo0406	*-1.1	2.8	**4.4**	similar to *B. subtilis* YyaH protein
lmo0444	*1.1	**-3.6**	-2.3	conserved hypothetical protein
lmo0469	*-1.5	*1.0	**3.7**	unknown
lmo0495	*1.1	**-7.3**	*-1.4	similar to transmembrane protein
lmo0498	*1.2	*-1.4	**-10.9**	similar to ribose 5-phosphate isomerase
lmo0536	*1.2	*-1.6	**-3.9**	similar to 6-phospho-beta-glucosidase
lmo0554	*1.3	*-1.2	**5.3**	similar to NADH-dependent butanol dehydrogenase
lmo0589	*-1.1	*1.5	**4.1**	unknown
lmo0611	*-1.0	*1.2	**3.6**	similar to acyl-carrier protein phosphodiesterase and NAD(P)H dehydrogenase
lmo0643	*-1.1	*1.1	**-3.8**	similar to putative transaldolase
lmo0655	*-1.2	*1.2	**4.2**	similar to phosphoprotein phosphatases
lmo0670	*-1.6	**5.7**	-1.2	unknown

lmo0672	*1.2	*1.7	**4.0**	similar to unknown protein
lmo0673	**4.3**	**4.4**	**5.8**	unknown
lmo0675	*1.4	**4.5**	*-1.1	similar to flagellar switch protein FliN
lmo0677	*1.7	**5.1**	2.9	similar to flagellar biosynthesis protein FliQ
lmo0678	2.5	*6.9	**6.7**	similar to flagellar biosynthetic protein FliR
lmo0679	**8.4**	**100.0**	**4.5**	similar to flagellar biosynthetic protein FlhB
lmo0680	**5.8**	**5.8**	*1.3	similar to flagella-associated protein FlhA
lmo0682	2.5	**9.6**	*-1.2	similar to flagellar hook-basal body protein FlgG
motA	**3.8**	**4.4**	2.0	similar to motility protein (flagellar motor rotation) MotA
motB	*4.7	**6.4**	*-1.3	similar to motility protein (flagellar motor rotation) MotB
lmo0687	**5.6**	**10.3**	*-1.2	unknown
lmo0688	**15.8**	1.3	2.4	similar to unknown protein
lmo0689	**5.5**	**13.1**	-2.1	similar to CheA activity-modulating chemotaxis protein CheV
flaA	**84.1**	**63.2**	*1.1	flagellin protein
cheY	**9.0**	**6.2**	**3.6**	chemotaxis response regulator CheY
cheA	**17.5**	**14.0**	2.0	two-component sensor histidine kinase CheA
lmo0693	**23.2**	**100.0**	2.0	similar to flagellar motor switch protein FliY C-terminal part
lmo0694	**14.5**	**25.5**	2.9	unknown
lmo0695	**20.0**	**16.7**	*2.1	unknown
lmo0696	**12.9**	**25.2**	3.2	similar to flagellar hook assembly protein
lmo0697	**30.9**	**29.7**	2.1	similar to flagellar hook protein FlgE
lmo0698	**19.8**	**26.3**	*1.2	weakly similar to flagellar switch protein
lmo0699	**100.0**	**100.0**	1.6	similar to flagellar switch protein FliM
lmo0700	**12.4**	**18.8**	1.9	similar to flagellar motor switch protein FliY
lmo0701	**5.8**	*4.5	3.1	unknown
lmo0702	**11.9**	**81.0**	3.3	unknown
lmo0703	**38.7**	**50.1**	**4.0**	unknown
lmo0704	**9.1**	**67.3**	2.2	unknown
lmo0705	**50.2**	**58.6**	**5.6**	similar to flagellar hook-associated protein FlgK
lmo0706	**14.3**	**34.7**	*1.8	similar to flagellar hook-associated protein 3 FlgL
lmo0707	**53.9**	**100.0**	**7.1**	similar to flagellar hook-associated protein 2 FliD
lmo0708	**28.3**	**34.0**	*1.4	similar to hypothetical flagellar protein
lmo0709	**28.1**	**37.2**	1.6	unknown
lmo0710	**20.7**	**12.0**	1.7	similar to flagellar basal-body rod protein FlgB
lmo0711	**18.7**	**9.0**	1.7	similar to flagellar basal-body rod protein FlgC
lmo0712	**18.7**	**43.4**	1.7	similar to flagellar hook-basal body complex protein FliE
lmo0713	**27.9**	**5.2**	2.9	similar to flagellar basal-body M-ring protein FliF
lmo0714	**15.5**	**13.5**	1.9	similar to flagellar motor switch protein FliG
lmo0715	**7.2**	3.2	*1.3	unknown
lmo0716	**11.1**	**3.7**	*1.7	similar to H+-transporting ATP synthase alpha chain FliI, flagellar-specific
lmo0717	**9.0**	*3.1	3.2	similar to transglycosylase
lmo0723	**9.3**	**45.6**	**7.6**	similar to methyl-accepting chemotaxis protein
lmo0724	**13.1**	**36.8**	**5.6**	similar to *B. subtilis* YvpB protein
lmo0736	*-1.0	*1.2	**-18.4**	similar to ribose 5-phosphate isomerase
lmo0813	*1.1	*-1.4	**-3.6**	similar to fructokinases
lmo0852	*1.4	*-1.5	**5.9**	similar to transcription regulator TetR/AcrR family

lmo0869	*1.1	**3.6**	*-8.7	unknown
lmo0903	*-1.2	1.2	**4.2**	conserved hypothetical protein
lmo0914	*1.4	**4.2**	*3.4	similar to PTS system, IIB component
lmo1042	*1.2	*1.4	**-5.9**	similar to molybdopterin biosynthesis protein MoeA
lmo1043	*1.1	*1.4	**-4.7**	similar to molybdopterin-guanine dinucleotide biosynthesis MobB
lmo1044	*1.5	1.9	**-4.2**	similar to molybdopterin converting factor, subunit 2
lmo1064	-1.8	*1.4	**3.7**	similar to membrane and transport proteins
lmo1123	*-1.3	*1.0	**3.9**	unknown
lmo1129	*-1.2	*-1.0	**-18.5**	similar to unknown proteins
lmo1150	*-1.1	2.1	**-5.1**	similar to *S. typhimurium* regulatory protein PocR
lmo1186	*-100	*-1.8	**-7.8**	similar to *E. coli* ethanolamine utilization protein EutH
lmo1202	*1.5	*1.1	**-3.7**	similar to anaerobic cobalt chelatase in cobalamin biosynthesis
lmo1254	*-1.1	*1.2	**-4.5**	similar to alpha,alpha-phosphotrehalase
lmo1256	*1.1	*1.2	**-5.1**	unknown
lmo1261	*-1.2	*1.1	**6.4**	unknown
lmo1266	*-1.3	*2.5	**11.2**	unknown
lmo1298	1.2	*-1.4	**4.1**	similar to glutamine synthetase repressor
lmo1310	*-1.2	*-1.6	**5.7**	similar to *E. coli* YbdN protein
lmo1369	*-1.2	*-1.0	**3.7**	similar to phosphotransbutyrylase
lmo1395	*-1.1	*1.3	**3.6**	similar to unknown protein
lmo1424	*-1.1	*1.4	**4.2**	similar to manganese transport proteins NRAMP
lmo1428	*-1.3	*-1.2	**3.5**	similar to glycine betaine/carnitine/choline ABC transporter (ATP-binding protein)
lmo1527	*-1.3	*-1.5	**3.9**	similar to protein-export membrane protein SecDF
lmo1536	*1.0	*1.1	**3.7**	similar to prephenate dehydratase PheA
lmo1545	*-1.3	*1.1	**4.5**	similar to cell-division inhibition (septum placement) protein MinC
comC	**-100.0**	**-100.0**	*7.1	similar to *B. subtilis* late competence protein ComC (type IV prepilin peptidase)
lmo1556	*-1.1	*-1.1	**3.6**	highly similar to porphobilinogen deaminases (hydroxymethylbilane synthase)
lmo1557	-1.3	*-1.2	**3.8**	highly similar to glutamyl-tRNA reductase
lmo1685	*-1.1	*1.1	**-4.3**	glutamate-1-semialdehyde aminotransferase
lmo1699	**3.7**	**36.3**	**6.9**	some similarities to methyl-accepting chemotaxis proteins
lmo1700	2.7	**4.1**	**9.7**	unknown
lmo1715	1.4	*1.5	**-4.0**	similar to hypothetical proteins
lmo1718	*1.1	*-1.1	**-3.9**	similar to putative outer surface protein
lmo1730	*-1.1	*1.7	**-8.3**	similar to sugar ABC transporter binding protein
lmo1732	*1.1	*1.4	**-3.7**	similar to sugar ABC transporter, permease protein
lmo1749	*-1.2	*-1.3	**8.5**	similar to shikimate kinase
lmo1755	*-1.1	*-1.3	**3.5**	glutamyl-tRNA(Gln) amidotransferase (subunit A)
lmo1790	*1.0	**5.7**	*1.9	similar to unknown proteins
lmo1810	*-1.1	*1.2	**4.8**	similar to unknown proteins
lmo1815	*-1.2	*1.1	**4.3**	similar to unknown protein
lmo1838	*-1.1	*-1.3	**4.0**	highly similar to aspartate carbamoyltransferase
lmo1852	*-1.1	-1.6	**-4.1**	similar to putative mercuric ion binding proteins
lmo1883	*1.0	-1.4	**-3.7**	similar to chitinases
lmo1912	*-1.3	*1.8	**5.6**	similar to unknown proteins (hypothetical sensory transduction histidine kinase)
lmo1931	*1.4	*1.4	**4.4**	similar to 2-heptaprenyl-1,4-naphthoquinone methyltransferase
lmo1933	*-1.1	*1.1	**4.0**	similar to GTP cyclohydrolase I

lmo1999	*1.1	*-1.5	**-35.8**	weakly similar to glucosamine-fructose-6-phosphate aminotransferase
lmo2001	*-1.7	*1.3	**-5.4**	similar to PTS mannose-specific enzyme IIC component
lmo2002	*1.3	*-1.2	**-8.1**	similar to PTS mannose-specific enzyme IIB component
lmo2004	*1.1	*-1.1	**-5.2**	similar to transcription regulator GntR family
lmo2046	*1.4	*-1.0	**4.1**	weakly similar to ketopantoate reductase involved in thiamin biosynthesis
lmo2058	*-1.1	*1.3	**7.1**	similar to heme O oxygenase
lmo2066	*-1.5	*1.3	**-4.2**	unknown
lmo2080	*-1.3	**-4.3**	*1.1	unknown
lmo2098	*1.1	-2.2	**-5.4**	similar to PTS system galactitol-specific enzyme IIA component
lmo2121	*-100	*-1.1	**-7.1**	similar to maltosephosphorylase
lmo2124	*1.0	*1.4	**-7.2**	similar to maltodextrin ABC-transport system (permease)
lmo2125	*1.1	*4.0	**-37.7**	similar to maltose/maltodextrin ABC-transporter (binding protein)
lmo2130	*-1.3	2.2	**4.1**	similar to unknown protein
lmo2135	*1.2	*1.2	**-4.1**	similar to PTS system, fructose-specific enzyme IIC component
lmo2137	*1.2	*-1.7	**-3.8**	similar to PTS system, fructose-specific enzyme IIA component
lmo2174	*-1.3	*3.8	**5.2**	similar to unknown proteins
lmo2212	*-1.4	*-1.1	**3.6**	similar to uroporphyrinogen III decarboxylase
lmo2232	-1.4	*-1.1	**3.5**	similar to unknown proteins
lmo2256	*1.1	*-1.2	**-4.0**	similar to unknown proteins
lmo2279	*-100	*-2.4	**-4.7**	holin [Bacteriophage A118]
lmo2280	*1.1	*-1.1	**-4.9**	protein gp23 [Bacteriophage A118]
lmo2281	*1.4	*-2.1	**-4.5**	protein gp22 [Bacteriophage A118]
lmo2282	1.3	*-1.7	**-5.9**	protein gp21 [Bacteriophage A118]
lmo2283	*-5.0	*-1.1	**-4.4**	protein gp20 [Bacteriophage A118]
lmo2284	*1.8	*1.1	**-5.1**	Protein gp19 [Bacteriophage A118]
lmo2285	*1.0	*-24.5	**-5.8**	Protein gp18 [Bacteriophage A118]
lmo2286	*-1.5	*1.1	**-3.8**	Protein gp17 [Bacteriophage A118]
lmo2288	*2.2	*1.8	**-3.6**	Protein gp15 [Bacteriophage A118]
lmo2290	1.3	*-1.1	**-4.4**	Protein gp13 [Bacteriophage A118]
lmo2291	*1.3	*1.3	**-4.3**	major tail shaft protein [Bacteriophage A118]
lmo2295	1.4	*-1.5	**-4.1**	protein gp8 [Bacteriophage A118]
lmo2297	*1.2	*-1.7	**-3.6**	putative scaffolding protein [Bacteriophage A118]
lmo2298	*-2.1	*-2.1	**-4.7**	protein gp4 [Bacteriophage A118]
lmo2299	*1.1	*-1.5	**-4.4**	putative portal protein [Bacteriophage A118]
lmo2300	*-1.5	*-1.2	**-7.5**	putative terminase large subunit from Bacteriophage A118
lmo2304	-2.1	*1.5	**-3.6**	bacteriophage A118 gp65 protein
lmo2314	*-1.2	*3.1	**-3.5**	unknown
lmo2324	*-1.5	*1.4	**-5.9**	similar to anti-repressor [Bacteriophage A118]
lmo2326	*-1.3	*-1.0	**-4.7**	similar to protein gp41 [Bacteriophage A118]
lmo2352	*-2.5	*-1.7	**5.5**	similar to LysR family transcription regulator
lmo2494	-1.7	**4.8**	*1.4	similar to negative regulator of phosphate regulon
lmo2585	1.3	*-1.2	**-3.7**	similar to *B. subtilis* YrhD protein
lmo2586	1.5	*1.4	**-3.7**	similar to formate dehydrogenase alpha chain
lmo2646	*-1.0	**4.3**	**-28.9**	unknown
lmo2647	*-1.2	**5.6**	**-17.1**	similar to creatinine amidohydrolase
lmo2648	*1.0	*-1.3	**-13.9**	similar to phosphotriesterase

lmo2650	*1.4	*3.9	**-11.7**	similar to hypothetical PTS enzyme IIB component
lmo2651	*1.2	**3.9**	**-10.2**	similar to mannitol-specific PTS enzyme IIA component
lmo2659	*1.4	**-5.7**	**-7.6**	similar to ribulose-phosphate 3-epimerase
lmo2660	*1.3	-1.9	**-7.1**	similar to transketolase
lmo2661	1.5	*-1.4	**-41.7**	similar to ribulose-5-phosphate 3-epimerase
lmo2662	*1.1	*-2.9	**-17.3**	similar to ribose 5-phosphate epimerase
lmo2663	*1.0	*1.2	**-33.9**	similar to polyol dehydrogenase
lmo2664	*-1.0	*-1.5	**-26.8**	similar to sorbitol dehydrogenase
lmo2665	*-1.1	*1.3	**-23.0**	similar to PTS system galactitol-specific enzyme IIC component
lmo2666	*3.7	*-1.0	**-20.5**	similar to PTS system galactitol-specific enzyme IIB component
lmo2667	*1.5	2.8	**-11.6**	similar to PTS system galactitol-specific enzyme IIA component
lmo2668	*-1.3	-2.0	**-13.2**	similar to transcriptional antiterminator (BglG family)
lmo2684	3.2	**47.2**	*-1.3	similar to cellobiose phosphotransferase enzyme IIC component
lmo2730	*-1.2	-2.4	**-3.6**	similar to phosphatase
lmo2731	*1.1	-2.3	**-4.2**	similar to transcription regulator (RpiR family)
lmo2742	*-1.0	-1.2	**-3.9**	unknown
lmo2752	*-1.3	**-4.1**	-1.6	similar to ABC transporter, ATP-binding protein
lmo2763	*-1.0	-6.2	**-3.9**	similar to PTS cellobiose-specific enzyme IIC
lmo2764	*1.3	**-6.0**	*-3.0	similar to xylose operon regulatory protein and to glucose kinase
lmo2773	*-1.3	-3.4	**-4.1**	similar to transcription antiterminator
lmo2818	*1.2	*-1.3	**-3.6**	similar to transmembrane efflux protein
lmo2848	*1.4	-2.2	**-3.9**	highly similar to L-rhamnose isomerase

* P-value greater than 0.01; bold-type indicates expression change greater than 3.5-fold with a P-value less than 0.01. Gene names correspond to the gene names on the ListiList server http://genolist.pasteur.fr/ListiList/. The functions of the encoded proteins are indicated according to the EGD-e genome annotation where possible [1]. Rosetta Resolver software analysis sets the maximal fold-change possible as 100 [2]. Only genes which yielded an expression change greater than 3.5-fold with a P-value less than 0.01 in at least one of the growth conditions are listed in the table.

References

1. Glaser P, Frangeul L, Buchrieser C, Rusniok C, Amend A, et al. (2001) Comparative genomics of *Listeria* species. Science 294: 849-852.
2. Hughes TR, Marton MJ, Jones AR, Roberts CJ, Stoughton R, et al. (2000) Functional discovery via a compendium of expression profiles. Cell 102: 109-126.
