# Supplementary material for: The MogR Transcriptional Repressor Regulates Nonhierarchal Expression of Flagellar Motility Genes and Virulence in Listeria monocytogenes
Source: PLoS Pathog. 2006 Apr 14;2(4):e30. doi: 10.1371/journal.ppat.0020030 (PMC1435793; doi:10.1371/journal.ppat.0020030)
Supplement: Supplementary file 2 [file ppat.0020030.st002.pdf]

Table S2. Putative MogR-regulated genes defined by genome-wide search for TTTT-N_5-AAAA sites in upstream sequences.

		J774, 37˚C		BHI, 37˚C		BHI, RT		
**Accession number**	**Position (bp)**	**Fold Change**	**P-value**	**Fold Change**	**P-value**	**Fold Change**	**P-value**	**Gene function**
lmo00004	140, 109	1.0	0.88908	-1.7	2.62E-06	1.6	0.07202	similar to *B. subtilis* YaaA protein
lmo00010	104, 74	-1.2	0.06273	-1.2	0.26415	1.7	0.05114	similar to mevalonate kinase
lmo00189	136, 116	-1.1	0.58281	-1.1	0.38322	1.3	0.16889	highly similar to *B. subtilis* Veg protein
lmo00675	150, 119	1.4	0.05607	**4.5**	0.00005	-1.1	0.6259	similar to flagellar switch protein FliN
lmo00690	132, 101, 80	**84.1**	0	**63.2**	3.36E-44	1.1	0.47534	flagellin protein (*flaA*)
lmo00691	124, 103, 72	**9.0**	4.64E-13	**6.2**	0.00095	**3.6**	4.09E-20	chemotaxis response regulator CheY (*cheY*)
lmo00703	117, 96	**38.7**	1.85E-16	**50.1**	6.25E-23	**4.0**	0.00001	unknown
lmo00723	98, 77, 56	**9.3**	6.68E-41	**45.6**	0	**7.6**	0	similar to methyl-accepting chemotaxis protein
lmo01013	168, 147	-1.0	0.97158	1.0	0.95848	1.2	0.14379	similar to conserved hypothetical proteins
lmo01014	235, 214	-1.4	0.03998	1.2	0.42587	3.0	3.73E-08	highly similar to glycine betaine ABC transporter (*gbuA*)
lmo01019	130, 109	-1.2	0.24261	1.3	0.39726	2.0	0.00003	similar to *B. subtilis* YitL protein
lmo01074	221, 201	-1.1	0.70105	1.3	0.3562	2.3	0.05055	highly similar to teichoic acid permease TagG
lmo01266	88, 67	-1.3	0.29896	2.5	0.10742	**11.2**	1.35E-22	unknown
lmo01648	89, 58	-1.0	0.87133	1.3	0.59553	1.8	0.07119	unknown
lmo01687	128, 107	-1.2	0.14102	-1.1	0.19832	1.4	0.00188	similar to hypothetical proteins
lmo01948	181, 150	-1.6	0.0001	-1.1	0.37668	-1.0	0.9712	similar to two-component response regulator ResD (*resD*)
lmo02142	84, 53	-1.2	0.44595	1.5	0.05834	-3.2	0.03729	unknown
lmo02143	172, 141	1.2	0.74517	2.3	0.11148	-1.9	0.09321	weakly similar to mannose-6-phosphate isomerase
lmo02165	142, 121	-1.9	0.00009	1.2	0.51474	2.3	0.00007	similar to transcription regulator CRP/FNR family
lmo02185	46, 25	-3.4	0.31371	8.3	1	2.0	0.7739	unknown
lmo02191	282, 252, 221	-1.4	0.00203	1.3	0.02575	1.9	0.00018	similar to unknown proteins
lmo02206	108, 87	-1.1	0.67469	-1.0	0.5747	1.2	0.69028	similar to endopeptidase ATP-binding chain B ClpB (*clpB*)
lmo02337	68, 47	-1.1	0.39406	-1.3	0.23786	-2.0	0.06151	similar to regulatory protein DeoR family
lmo02338	180, 160	-1.0	0.78353	-1.2	0.12752	-1.5	0.00557	aminopeptidase C
lmo02378	238, 218	-1.0	0.81321	1.3	0.4245	1.7	0.0002	similar to proteins involved in resistance to cholate

The ListiList server was searched for genes containing a minimum of two TTTT-N_5-AAAA sites (allowing one mismatch/site) spaced 7-9 bp or 17-19 bp apart (1 or 2 helix turns, respectively) within 300 bp of upstream sequences. Accession numbers correspond to those given by the ListiList server http://genolist.pasteur.fr/ListiList/. Fold expression change is given for MogR-negative *L. monocytogenes* relative to wild-type. Candidate genes that were found by microarray analysis to be MogR-regulated (>3.5-fold, $P < 0.01$) are underlined with the fold expression change in bold. The position refers to the location of an upstream TTTT-N_5-AAAA site relative to the translational start codon of the gene listed. The functions of the encoded proteins are indicated according to the EGD-e genome annotation where possible, and gene names with a non-*lmo* suffix are indicated in parentheses [1]. Fold change and P-values are derived from Rosetta Resolver analysis of microarray data generated under the conditions listed.

References

1. Glaser P, Frangeul L, Buchrieser C, Rusniok C, Amend A, et al. (2001) Comparative genomics of *Listeria* species. Science 294: 849-852.
