# Supplementary material for: The MogR Transcriptional Repressor Regulates Nonhierarchal Expression of Flagellar Motility Genes and Virulence in Listeria monocytogenes
Source: PLoS Pathog. 2006 Apr 14;2(4):e30. doi: 10.1371/journal.ppat.0020030 (PMC1435793; doi:10.1371/journal.ppat.0020030)
Supplement: Supplementary file 3 [file ppat.0020030.st003.pdf]

Table S3. ***Listeria monocytogenes*** **and** ***Escherichia coli*** **strains.**

Listeria monocytogenes

**Strain**	**Genotype and relevant features**	**Strain Designation**	**Reference**
DH-L478	Wild-type *L. monocytogenes* strain EGDe	wild-type	M. Loessner
DH-L975	Tn917 insertion in *flaA* in EGDe		[1]
DH-L1042	*flaA* in-frame deletion in EGDe	Δ*flaA*	[1]
DH-L1156	*mogR* in-frame deletion in EGDe	Δ*mogR*	[1]
DH-L1179	Tn917 insertion in *flaA* in DH-L1156		[1]
DH-L1248	*flaA* in-frame deletion in DH-L1156	Δ*mogR* Δ*flaA*	This study
DH-L1273	*degU* in-frame deletion in EGDe	Δ*degU*	This study
DH-L1274	*degU* in-frame deletion in DH-L1156	Δ*mogR* Δ*degU*	This study
DH-L1275	*degU* in-frame deletion in DH-L975		This study
DH-L1276	*degU* in-frame deletion in DH-L1179		This study

Escherichia coli

**Strain**	**Genotype and relevant features**	**Reference**
DH-E113	BL21(DE3)	Novagen
DH-E122	pET29b in BL21(DE3)	Novagen
DH-E123	pCON1 in JM109	[2]
DH-E182	XL1-Blue {F’ *proAB* $lacI^q$ Δ(*lacZ*)M15 Tn10} *recA1 endA1 gyrA96 thi-1 hsdR17 supE relA1 lac*	Stratagene
DH-E1272	pCON1/Δ*degU* in XL1-Blue	This study
DH-E1334	pET29b derivative with *mogR* in XL1-Blue	This study
DH-E1335	pET29b derivative with *mogR* in BL21(DE3)	This study

References

1. Gründling A, Burrack LS, Bouwer HG, Higgins DE (2004) *Listeria monocytogenes* regulates flagellar motility gene expression through MogR, a transcriptional repressor required for virulence. Proc Natl Acad Sci USA 101: 12318-12323.
2. Freitag NE (2000) Genetic tools for use with *Listeria monocytogenes*. In: Fischetti VA, Novick RP, Ferretti JJ, Portnoy DA, Rood JI, editors. Gram-positive pathogens. Washington, D.C: ASM Press. pp. 488-498.
