# Supplementary material for: The MogR Transcriptional Repressor Regulates Nonhierarchal Expression of Flagellar Motility Genes and Virulence in Listeria monocytogenes
Source: PLoS Pathog. 2006 Apr 14;2(4):e30. doi: 10.1371/journal.ppat.0020030 (PMC1435793; doi:10.1371/journal.ppat.0020030)
Supplement: Supplementary file 4 [file ppat.0020030.st004.pdf]

Table S4. Oligonucleotides used in this study.

Number	Sequence	Site[a]
348	ACGCGATATCCTTCTCTATATGCTTTATTATATCTTC	*EcoRV*
376	ATCTTTCACGATATTCTTAATCATCGTACGCATGAACATCGC	–
462	GGAATTCCATATGCCTAAATCAGAAATAAGAAAATT	*NdeI*
463	CCGCTCGAGCATTTGTTTATAATTTTCTTTG	*XhoI*
464	GCTCTAGAATTCAACAATAGTGGCTAG	*XbaI*
465	CCGTGCTTGATTGCCGTTACGCGAAACAACTGATGATC	–
466	GGGGTACCCTTAATTCAAATGTTACTTCTGG	*KpnI*
467	GATCATCAGTTGTTTCGCGTAACGGCAATCAAGCACGG	–
489	TCCCCCCGGGACTTTTCTGTTCAAACACTT	*XmaI*
490	TCCCCCCGGGCAGAGCCAATGTTAAGTTGG	*XmaI*
492	CAATATTTTTCATGATAGTTGCCAT	–
493	AATTGAAACATTATTCATCCTT	–
494	GCCGATATTCACTAATTATCATAACG	–
495	ACGATTTTTAATTAAATTCAC	–
496	AGCTTAATTTTTCTATATACCCAT	–
497	CCGCATTAACATTCATTAAAAAAACC	–
498	TTACATTTTTAATAAAAAACATTTGATTTTTTAAAAAAATGAAGAT	–
499	ATCTTCATTTTTTTAAAAAATCAAATGTTTTTTATTAAAAATGTAA	–
500[b]	TTACATTTTTAATAAAAAACATTTGAT**AA**TTTAAA**TT**AATGAAGAT	–
501[b]	ATCTTCATT**AA**TTTAAA**TT**ATCAAATGTTTTTTATTAAAAATGTAAGAT	–
502[b]	TTACATTTTT**TT**TAAAAAACA**CC**TGATTTTT**AT**AAAAAATGAAGAT	–
503[b]	ATCTTCATTTTTTA**TA**AAAATCA**GG**TGTTTTTTA**AA**AAAAATGTAA	–

a. The indicated restriction endonuclease site is underlined within the oligonucleotide sequence.
b. Deviations from the wild-type sequence are in bold within the oligonucleotide sequence.
